# Can cancer patients assess the influence of pain on functions? A randomised, controlled study of the pain interference items in the Brief Pain Inventory

**DOI:** 10.1186/1472-684X-6-2

**Published:** 2007-03-09

**Authors:** Guri Stenseth, Marit Bjørnnes, Stein Kaasa, Pål Klepstad

**Affiliations:** 1 Dept. of Cancer Research and Molecular Medicine, Norwegian University of Science and Technology, Trondheim, Norway; 2 Dept. of Circulation and Medical Imaging, Norwegian University of Science and Technology, Norway; 3 Department of Oncology, St. Olavs University Hospital, Trondheim, Norway; 4 Department of Anaesthesia and Acute Medicine, St. Olavs University Hospital, Trondheim, Norway

## Abstract

**Background:**

The Brief Pain Inventory (BPI) is recommended as a pain measurement tool by the Expert Working Group of the European Association of Palliative Care. The BPI is designed to assess both pain severity and interference with functions caused by pain. The purpose of this study was to investigate if pain interference items are influenced by other factors than pain.

**Methods:**

We asked adult cancer patients to complete the original and a revised BPI on two study days. In the original version of the BPI the patients were asked how, during the last 24 hours, pain has interfered with functions. In the revised BPI this question was changed to how, during the last 24 hours, these functions are affected in general. Heath related quality of life was assessed at both study days applying the European Organization for Research and Treatment of Cancer quality of life questionnaire.

**Results:**

Forty-eight of the 55 included patients completed both assessments. The BPI pain intensities scores and the health related quality of life scores were similar at the two study days. Except for mood this study observed no significant distinctions between the patients' BPI interference items scores in the original (pain influence on function) and the revised BPI (function in general). Seventeen patients reported higher influence from pain on functions than the total influence on function from all causes.

**Conclusion:**

We observed similar scores in the original BPI interference scores (pain influence on function) compared with the revised BPI interference scores (decreased function in general). This finding might imply that the BPI interference scale measures are partly responded to as more of a global interference measure.

## Background

Pain is a one of the major distressing symptoms experienced by patients with a malignant disease. To monitor the efficacy of pain treatments it is important to have valid methods for pain measurements. Such pain assessments should be easily completed and communicated. It is generally recognised that pain measurement should use methods based on patients' self-report. Numeric scales are easy to understand and easy to score and have for these reasons been endorsed for use in both in cancer clinical trials and trials of chronic non-malignant pain [1,2].

Cleeland and colleagues developed the Brief Pain Inventory (BPI), a questionnaire designed to assess both pain severity and the interference from pain on seven areas of daily life [3]. The BPI is self-administrated and easily understood, and is translated and validated in several languages [4-8]. The BPI is recommended as a research pain measurement tool by the Expert Working Group of the European Association of Palliative Care [1], and it is one of the most widely used multi-dimensional measures for cancer pain. Studies using the BPI have observed that the BPI interference scores are higher in patients with deteriorated functional performance compared with scores in patients with normal or near normal functional performance [6,7]. This difference was not explained by higher pain ratings in patients with lower functional performance. This lack of relationship to pain intensity questions cancer patients' ability to report the influence on function from pain without a bias from decreased function caused by other factors. Given the ubiquitous presence of BPI in cancer pain research it is important to consider factors that may constrain the validity of the interference scale. To investigate whether pain interference items are answered independently of decreased function due to other causes than pain we compared the original BPI with a modified version. The modified version asks about degree of functions without specifying the cause of the impaired functions.

## 2. Methods

### 2.1. Patients

We included 55 in-patients at the Department of Oncology at St. Olavs Hospital, a 400-bed tertiary university hospital. Patients, who had an established diagnosis of cancer and who were at least 18 years of age, were eligible. Patients with cognitive failure, with planned hospitalisation less than 4 days or with scheduled surgery in the study-period were not included. All patients were naive to the BPI questionnaire.

### 2.2. Asssements

Demographic data were collected from the patients' hospital records. We registered age, gender, malignant diagnosis, date for diagnosis, anticancer treatment (former and ongoing) and all medications on both observations. The patients' functional status was assessed using the Karnofsky score [9].

To provide information regarding health related quality of life (HRQOL) we used the European Organization for Research and Treatment of Cancer (EORTC) QLQ-C30 questionnaire [10]. It has high reliability and validity in different groups of cancer patients [10,11] and the test-retest reliability is optimal [12]. The questionnaire consists of 30 items. It is composed of five functional scales (physical, role, emotional, cognitive, social), three symptom scales (fatigue, pain, nausea/vomiting), and eight single items (global health, global quality of life, dyspnea, appetite loss, sleep disturbance, constipation, diarrhea, financial impact of the disease and treatment).

### 2.3. BPI questionnaire

The first part of the BPI measures pain severity using four different 11-point numeric scales anchored by 0 representing "no pain" and 10 being "pain as bad as you can imagine". Patients are instructed to rate their pain now and worst for the last 24 hours, least for the last 24 hours and average pain. The second part of the BPI measures how pain interferes with general activity, mood, walking, normal work, relationships with others, sleep and enjoyment of life. Similar to pain severity each functional item is ranked on an 11-point numeric scale, where 0 represents "Does not interfere" and 10 denotes "completely interferes". The sum of the scores of the pain intensity items represents the summed pain intensity score and the sum of the scores on the pain interference items represent the summed interference score. Because BPI has no validated method for handling missing values the summed scores were not calculated for patients with missing values.

This study compared the original BPI with a revised version. In the original BPI the patients are asked to "Circle the one number that describes how, during the last 24 hours, pain has interfered with your general activity, mood, walking ability, normal work, relations with other people, sleep or enjoyment of life". In the revised BPI, designed for this study, this question was changed to "Circle the one number that describes how, during the last 24 hours, these functions are disturbed". In both versions of the BPI this question was written in bold letters. The numeric rate scale (0–10) and the anchor descriptive statements were identical in the original and in the revised BPI.

### 2.4. Procedure

**Figure 1:**
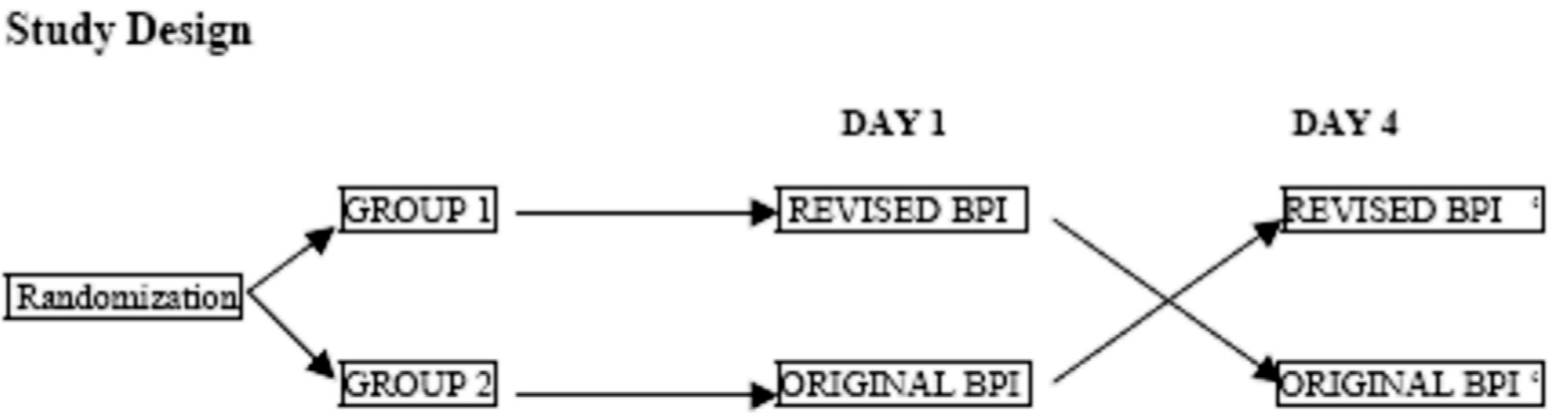
Study design.

The participants were asked to complete the BPI (original or revised version) and the EORCT QLQ-C30 on both study days. The sequence of the BPI questionnaires was randomised and an equal number of the patients received the original and the revised version of the BPI at the first study day (fig. 1). In order to not let the EORTC QLQ-C30 procedure influence the patients BPI responses, the EORTC questionnaire was answered after the completion of the BPI questionnaires. The randomization procedure was performed by the office for Clinical Cancer Research, Norwegian Health Region IV. The interval between the two study days was four days. The questionnaires were delivered to and collected directly from the patients. This procedure ensured that all questionnaires were returned. The majority of the BPI questionnaires were completed by the patients. However, because of practical difficulties with writing (e.g. supine position, attached to intravenous lines) some patients gave verbal responses to the questions, which were recorded in the questionnaires by one of the study investigators. All interviewers received training in performing a standard interview technique. They were instructed to clarify, but not to amplify the questions in response to the patients' inquiries.

### 2.4 Ethics

The Regional Committee for Medical Research Ethics, Health region IV, Norway, approved the study, and all patients gave their oral and written informed consent before inclusion into the study.

### 2.5. Statistical evaluation

In the sample size calculation for the study we used a clinical difference of interest of two points on the 11-point numeric rate scale [13]. The population standard deviations observed in the Norwegian BPI validation paper were approximately 2.4 for the BPI interference scores [7]. As the present study compared two observations obtained in each participant it is correct to use the within standard deviation for sample size calculations. The within standard deviation is not known, but must per definition be less than the combined standard deviation. As a conservative estimation of SD_within _for use in this sample size calculation we used a SD_within _of 1.2. Thus, we assumed that half of the variation is due to within subjects variation and half of the variation is due to between subjects variation. For paired observations, significance level of 0.05, power of 0.90 and two-sided tests the appropriate sample size was 27 paired observations [14]. Allowing for drop-outs between the observations we planned for a study with a minimum number of 50 participants.

Statistical comparisons between the trial days were performed using paired Student t-test for continuous data and Wilcoxon-test for categorical variables.

The statistical software SPSS version 11.02 for Windows was used throughout the analyses.

## 3. Results

### 3.1. Patient characteristics

We approached 65 patients who met the criteria for inclusion in the study. Ten patients refused to participate in the study. Of the 55 patients who were included into the study seven patients did not answer the second scheduled administration of the questionnaires (discharged from the hospital n = 2, patient felt too exhausted n = 4, patient died n = 1).

The patient sample consisted of 24 males and 31 females, with a mean age of 66 (range; 29–84) years. The most prevalent diagnoses were breast cancer (31%), colorectal cancer (18%), malignant lymphoma (11%) and lung cancer (9%). Sixty percent of the patients had confirmed cancer metastases. The Karnofsky score ranged from 30 to 100, with a mean score of 82. Twenty-four patients had received anticancer treatment prior to inclusion (radiotherapy n = 9, surgery n = 28, anti-hormonal therapy n = 10, chemotherapy n = 17), whereas 23 of the patients received anticancer treatment during the survey (radiotherapy n = 21, anti-hormonal therapy n = 11, chemotherapy n = 9).

Sixteen percent of the patients received regular scheduled analgesics (opioid analgesics n = 8, NSAIDs n = 3, paracetamol n = 2), whereas 24 percent of the patients used analgesics when needed.

### 3.2. Health related quality of life

We observed no statistical significant differences between the trial days in the EORTC QLQ-C30 pain scores (Table 1). The patients reported significantly higher fatigue, higher sleep disturbance and lower cognitive function scores at trial day two. The absolute difference in mean scores were 5.3, 9.2 and 3.8 for fatigue, sleep disturbance and cognitive function, respectively. The other EORTC QLQ-C30 scores were similar between the two study days (table 1). None of the observed statistical significant differences were higher than 10 which is the difference proposed by Osoba et al. to represent a clinical difference of interest (Osoba et al., 1998).

**Table 1:** EORTC QLQ-C30 Health related quality of life scores

	Day 1 Mean value (SD)	Day 4 Mean value (SD)	p-value (2 tailed paired samples test)
**Functional scores**			
Cognitive function	91,0 (14,6)	87,2 (21,0)	0,04
Emotional function	77,4 (23,4)	75,6 (25,2)	0,23
Physical function	61,4 (28,3)	59,6 (28,0)	0,42
Role function	49,0 (41,0)	43,8 (39,1)	0,18
Social function	41,0 (36,5)	42,4 (36,5)	0,63
			
**Symptom scores**			
Nausea and vomiting	10,8 (22,1)	9,0 (15,4)	0,53
Pain	38,9 (37,9)	41,7 (33,0)	0,39
Appetite	29,7 (38,6)	24,6 (35,4)	0,28
Constipation	25,7 (35,9)	27,1 (37,4)	0,69
Dyspnea	32,6 (38,6)	29,2 (36,1)	0,28
Fatigue	38,9 (29,8)	44,2 (28,9)	0,03
Sleep	19,9 (29,2)	29,1 (31,6)	0,04
Financial	4,9 (13,7)	4,2 (13,1)	0,74
			
Quality of life	56,0 (27,5)	53,1 (27,4)	0,35

### 3.3. BPI

Seven of the completed BPI questionnaires had one or two missing items. As shown in Table 2 the patients in general reported low intensities of pain. The pain interference scores in the modified version were not significantly different compared to those in the original BPI (Table 2), except for mood interference, which was statistically significantly higher in the modified BPI (Table 2). The summed interference score (the sum of all seven BPI interference items was equal in the revised and the original BPI for two patients, 26 patients had higher summed interference score in the revised BPI, and 17 patients had higher summed interference score in the original BPI.

**Table 2:** Scores at the original BPI and at the revised BPI

	Original BPI mean value (SD)	Revised BPI mean value (SD)	p-value (2-tailed paired samples test)
**Severity items**			
3. Pain at its worst	2,9 (3,0)	3,0 (3,3)	0,768
4. Pain at its least	0,3 (0,7)	0,7 (1,4)	0,058
5. Pain on the average	1,9 (1,9)	1,9 (2,2)	0,884
6. Pain right now	1,1 (1,7)	1,4 (2,3)	0,418
Summed pain intensity score	6,2	7,0	0,466
			
**Interference items**			
9. General activity	3,4 (3,8)	4,5 (3,7)	0,055
10. Mood	1,4 (2,5)	2,5 (3,1)	0,022
11. Walking ability	2,6 (3,6)	3,4 (3,8)	0,077
12. Normal work	3,6 (3,9)	4,6 (4,0)	0,140
13. Relations with other people	1,2 (2,5)	1,6 (2,4)	0,427
14. Sleep	1,7 (2,7)	2,0 (2,9)	0,425
15. Enjoyment of life	1,9 (2,9)	2,1 (2,6)	0,556
Summed interference score	16,6	20,6	0,093

## 4. Discussion

We observed similar scores in the original BPI interference scores (pain influence on function) compared with the revised BPI interference scores (decreased function in general). This finding suggests that it is difficult for patients to interpret the contribution from pain on functional limitations. This finding was further emphasized by that 17 patients reported a higher summed interference scores from pain interference on functions than the total summed interference on functions from all causes This observation means that some patients interpret the influence from pain on function as higher than the total reduction of function, a result that demonstrates an inability for these patients to decipher the specific influence from pain on function. The finding in this study also is in accordance in previous studies showing that pain interference scores are in general higher in patients with decreased all over functional performance [6,7].

In our study only about 40 % of the patients received analgesics and the pain ratings were generally low. These observations show that the majority of patients included in this study did not suffer from severe pain. Despite the reports of low intensities of pain several patients reported that pain influenced functions. This finding of pain interference in patients without any major pain further demonstrates the inability to deline pain influence on functions from functional limitations related to other causes. However, because of low pain intensities the observations in this study can not be generalized to patients with severe cancer pain. Furthermore, the patients in this study had in general a high level of function. Thus, a study including patients with more pain and more complex symptoms burden is indicated in order to study the BPI measure of influence on function on pain in patients with more advanced cancer disease. Furthermore, new studies should investigate if BPI validity changes in studies including patients with non-malignant chronic pain.

In studies comparing two different versions of a questionnaire it is critical to choose an ideal time interval between the paired observations. A short time interval gives a risk for recall bias, while a long interval is associated with a risk for that change in scores are associated with fluctuations in the patients symptoms intensities and not related to differences in the questionnaires. In previous studies on test/retest properties of questionnaires the time intervals between assessments have ranged from 2 to 14 days [12]. We choose a 4-day interval between the two assessments because a longer interval could be expected to be associated with detoriated general health. To control that health related quality of life was stable during the study period all patients also completed a general HRQOL questionnaires on both study days. The results from these ratings showed that the patients' functional scores were similar in respect to clinically significant differences on both study days. Furthermore, in order to protect against a systematic error caused by changes in functions over time we randomized the patient to receive the original and the revised BPI in different sequences.

The ratings of pain in the BPI precede the interferences scales. Therefore, it could be argued that pain may be the referent point for the patients' judgment about interference. Also it may be argued that in the context of the BPI pain intensity items, the patients may rate pain related interference in both versions of the questionnaire. However, for the aim of this study, to study the pain interference items of the BPI, the questionnaire has to be administered in the exact form as it is used in clinical studies. To omit the pain items and hereby doing a major change in the BPI design, would render the study invalid with respect to investigating the psychometric properties of the BPI questionnaire. If the interference items included in the BPI would perform different if not preceded by the pain items is not studied.

This study does not answer why patients have difficulties with reporting the specific influence from pain on functions. One potential explanation is that reduced cognitive function decreases the patients' ability to analyze and report the differential influence from different causes on functional ability. For patients included into this study, however, the cognitive function assessed by the EORTC QLQ-C30 cognitive score was comparable to the cognitive function reported in the general Norwegian population [15]. There are several factors besides cognitive function, which may influence the patients' ability to interpret the contribution of pain to functional limitations. Potential factors are cultural background, age, intellectual abilities, educational background and training in answering questionnaires. At the present the basic concept about individuals' ability to interpret causes that explain the self experienced functional limitations is not completely understood.

## 5. Conclusion

This study observed similar scores in the original BPI interference scores (pain influence on function) compared with the revised BPI interference scores (decreased function in general). This finding might imply that cancer patients' ability to interpret the contribution from pain to loss of function is limited.

## Competing interests

The author(s) declare that they have no competing interests.

## Authors' contributions

GS, MB, SK and PK all participated in the design of the study, data analyses and drafted the manuscript. PK conceived the study and performed the statistical analyses. All authors have approved the final manuscript.

## Pre-publication history

The pre-publication history for this paper can be accessed here:


